# The Role of Vitamin C and Vitamin D in the Pathogenesis and Therapy of Periodontitis—Narrative Review

**DOI:** 10.3390/ijms24076774

**Published:** 2023-04-05

**Authors:** Łukasz Ustianowski, Klaudia Ustianowska, Klaudia Gurazda, Marcin Rusiński, Piotr Ostrowski, Andrzej Pawlik

**Affiliations:** Department of Physiology, Pomeranian Medical University in Szczecin, 70-111 Szczecin, Poland

**Keywords:** periodontitis, vitamin C, vitamin D, diet therapy, periodontal disease, tooth loss

## Abstract

Periodontitis is a common disorder affecting the bone and soft tissues of the periodontal complex. When untreated, it may lead to severe mobility or even loss of teeth. The pathogenesis of periodontitis is complex, with crucial factors being chronic inflammation in gingival and periodontal tissues and oral microbiome alterations. However, recent studies highlight the alleged role of vitamins, such as vitamin C (VitC) and vitamin D (VitD), in the development of the disease. VitC regulates numerous biochemical reactions, but foremost, it is involved in synthesizing collagen. It was reported that VitC deficiency could lead to damage to the periodontal ligaments. VitC supplementation improves postoperative outcomes in patients with periodontitis. VitD is a steroid derivative that can be produced in the skin under ultraviolet radiation and later transformed into an active form in other tissues, such as the kidneys. VitD was established to decrease the expression of proinflammatory cytokines in gingiva and regulate the proper mineral density of teeth. Moreover, the supplementation of VitD was associated with better results in the nonsurgical treatment of periodontitis. In this review, we summarize recent knowledge on the role of vitamins C and D in the pathogenesis and treatment of periodontitis.

## 1. Introduction

Periodontitis is a common disease that is characterized by the developing destruction of the alveolar bone complex and soft tissues of the periodontal complex. It occurs in roughly 47% of the population. Periodontitis may cause mobility and loss of teeth or implants [1]. The overall pathogenesis of the process is an interaction between dysbiotic bacteria creating a plaque biofilm and irregular immune responses in the gingival and periodontal tissues [2]. Persistent inflammation, including the dysbiosis of the supra- and subgingival biofilm, are fundamental in the beginning and progression of periodontitis [3]. Continuous inflammation in periodontal tissue results in the destruction of connective tissue and alveolar bone structure. Furthermore, the oral commensal bacteria and pathobionts play a significant role in contributing to periodontitis by interfering with major pathogens, such as *P. gingivalis*, *T. forsythia*, and *A. actinomycetemcomitans* [4]. Periodontal inflammation and dysbiosis of microbiota are not only caused by the lack of oral hygiene but also by environmental components, for example, poor diet and psychological stress [5,6].

Periodontitis is a disease caused by a local immune response against a microbial infection taking place in the periodontal tissue, aiming to eliminate the causative agents. The initial inflammation in the periodontal tissues, which is a physiological defense in response to a pathogen, can turn into a chronic inflammatory process leading to activation of the immune system. However, plaque formation is a way for bacteria to potentially slow down the immune response and provide suitable conditions for other anaerobic bacteria, further increasing the pathogenicity of microorganisms in plaque. This results in the release of several mediators such as proinflammatory cytokines, chemokines, and metalloproteinases, which cause damage to periodontal tissues. If left untreated, the gradual resorption of attachment leads to tooth loosening and even tooth loss.

Recent studies indicate that oxidative stress is also involved in the pathogenesis of periodontal disease. It appears that oxidative stress may influence the development of chronic inflammation observed in periodontal tissues of patients with periodontitis. Associated with antioxidant stress, an increase in oxidants leads to damage to cell components. In the cell, there is damage to proteins and oxidation of lipids. Elevated amounts of reactive oxygen species (ROS) were found in inflamed periodontal tissues; in addition, serum levels of reactive oxygen metabolites and biomarkers of lipid peroxidation, such as malondialdehyde, were increased in patients with periodontal disease. Thus, it seems that oxidative inflammatory stress may be one of the likely causes of periodontal disease pathways [5,6]. Recent studies propose the role of various nutrients and vitamins, such as vitamin C (VitC) and vitamin D (VitD), in the development of periodontal disease.

VitC, also known as L-ascorbic acid, is a substance that cannot be synthesized by people; therefore, it should be provided with a proper diet [7]. About 90% of the daily requirement comes from vegetables and fruits, which are excellent sources of this vitamin [8]. The National Institutes of Health recommends a daily dose of VitC for adults: 75 mg for women and 90 mg for men. Scurvy, a potentially fatal disease, is caused by a heavy deficit of VitC [9]. Its symptoms consist of weak collagenous and connective structures, difficult wound healing, loosening of teeth, impaired immunity, and increased risk of severe infections such as pneumonia [10]. It is connected with collagen synthesis, which is a vital agent providing structural strength to connective tissue [11]. Typical symptoms of deficiency are muscle weakness, petechial hemorrhaging, spontaneous ecchymoses, anemia, lethargy, lassitude, and irritability. The disease may be lethal due to dangerous complications, such as a cerebral or myocardial hemorrhage [12]. As for the periodontal tissues, symptoms include pain in the gums, swelling, and bleeding resulting from the fragility of the vessels, which ultimately may lead to tooth loss [12]. Stabilization of the collagen tertiary structure is also dependent on the action of VitC [13].

The immune system is also modulated by many VitC activities. Due to the fact that this substance is a very effective antioxidant, it can protect essential biomolecules from damage by toxins, pollutants, and oxidants by donating electrons [14]. Furthermore, VitC has a significant impact on neutrophil function, stimulating its migration toward the infection and increasing phagocytosis and microbial killing. It also promotes the apoptosis of neutrophils and their removal by macrophages [15].

VitC is also involved in the process of synthesis of catecholamine hormones and amidated peptides, such as vasopressin, by being a cofactor for the hydroxylase enzymes. These hormones are crucial for the cardiovascular response to heavy infection [16].

VitD is synthesized from 7-dehydrocholesterol during a photochemical reaction and is further hydroxylated in the liver to 25-hydroxyvitamin D3 (25(OH)D3). This is the main plasma VitD, which is a biologically active metabolite that maintains the balance of calcium and phosphorus concentrations in the blood by regulating their absorption in the intestines and reabsorption in the kidneys. VitD is found in oily fish meat and fish oil, although its prevalence in other types of food is relatively low [17]. VitD has many significant physiological functions [18]. In addition, it has been shown that VitD has a direct and indirect impact on bone metabolism, which is vital in preventing osteoporosis, rickets, and fractures in the aging skeleton [19,20]. A meta-analysis carried out by Bischoff-Ferrari et al. showed that there is a connection between VitD supplementation and fracture prevention [21]. Moreover, it is an essential factor in preventing and helping in the treatment of diseases caused by impaired immune homeostasis [22]. VitD receptors have been found on monocytes, macrophages, neutrophils, and dendritic cells. They have been shown to be involved in the release of compounds with antimicrobial activity. This affects immune system function and is an important part of the defense mechanisms against bacterial infections in the oral cavity [22].

VitD is also crucial in metabolic diseases, such as obesity, diabetes, and metabolic syndrome. The level of 25(OH)D is commonly lower in obese patients, although VitD administration may improve or prevent the progression of diabetes [23,24,25]. Cardiovascular disease might be intensified by a low level of 25(OH)D as well, and the deficiency of VitD has a connection with cardiomyopathy [26,27]. Furthermore, recent studies showed that VitD may prevent cancer progression or inhibit the development of new metastases [28]. Serum levels of VitD are also crucial for oral health. VitD deficiency and impaired synthesis may cause many oral diseases and disorders, such as alteration of tooth mineralization in children, gingivitis, and periodontitis. Some oral pathologies, such as certain oral cancers or osteonecrosis of the mandible, can also be associated with VitD deficiency [29].

The results of previous studies indicated that vitamins C and D have a significant effect on the proper functioning of periodontal tissues and the development of periodontal disease when they are deficient. Currently, there are a number of studies examining the effectiveness of these vitamins in the treatment of periodontitis. The purpose of this review is to summarize studies evaluating the role of vitamins C and D in the development and therapy of periodontitis.

## 2. Vitamin C in Periodontitis

L-ascorbic acid is a nutrient essential for every human being. The lack of L-gulono-1,4-lactone oxidase in the human body results in the inability to produce this molecule [12]. VitC has a broad spectrum of influence on metabolic processes in the human body. The crucial functions are the regulation of collagen, corticosteroids, neurotransmitter synthesis, iron absorption, and immune system reactions [12]. VitC enables the formation of intermolecular collagen cross-links, which strengthens the covalent bonds between the lysine and hydroxylysine molecules of the adjacent tropocollagen molecules. In the absence of hydroxylation, the synthesis of procollagen decreases [11], and it probably undergoes faster degradation [7]. As VitC is an essential element of the collagen stabilization process, its deficiency may lead to the instability of this protein, which is associated with the weakening of the periodontal ligaments and, consequently, the loss of teeth. Moreover, L-ascorbic acid plays a vital role in endothelial cell function and support. Research showed that it stimulates the proliferation of endothelial cells [30,31], probably due to its ability to increase the synthesis of type IV collagen [32]. The effect of VitC on various proinflammatory cytokines highlights its promising role as a biomarker or additional therapeutic option. Mikirova et al. showed that high doses of intravenous VitC can cause a decrease in proinflammatory cytokines and C-reactive protein in patients with cancer [33]. Moreover, Helmersson et al. reported that low dietary intake of ascorbic acid is associated with increased inflammatory and oxidative stress [34]. This could be the basis for starting research to establish the occurrence of a similar association in patients with periodontitis. Despite VitC’s antioxidant properties, in high doses, it has an oxidizing effect. Studies report that high doses of VitC may have a pro-oxidative effect and can even selectively kill cancer cells [35]. However, the anticancer effect of this substance needs to be better understood. Thanks to its influence on the thickness of the cell walls through better-quality collagen fibers, VitC increases the strength of the vessels. Furthermore, VitC stimulates the migration of fibroblasts in the skin and the proliferation of keratinocytes, which may positively affect wound healing that could result from gingivitis having an impact on reducing the inflammatory process within the gums [36,37]. In summary, previous studies indicated that VitC exerts anti-inflammatory effects in periodontal tissues by inhibiting oxidative stress and synthesis of proinflammatory mediators, inhibits the development of bacterial infection by increasing the phagocytic capacity of neutrophils, and increases collagen synthesis and strength of the vessels, leading to better tissue regeneration.

In this article, we will focus on the potential role of VitC in periodontitis. Periodontitis is associated with the destruction of the tissues surrounding the periodontium, namely the periodontium, bone, and gingiva. A study shows that the health of periodontal ligament cells (PDLcs) significantly impacts the development of this medical condition. As a result of oxidative stress, free radical damage to the cells occurs, and periodontitis progresses. Wu et al. conducted a study evaluating the cytotoxic effect of hydrogen peroxide (H_2_O_2_) and the antioxidant function of VitC in PDLcs. H_2_O_2_ induces apoptosis in PDLcs; however, the addition of VitC partially inhibited this process. They showed that VitC action as a reductant might have a protective effect on periodontal ligaments and could be a potential substance for periodontitis treatment [38]. Ahuja and others described that VitC injection along with microneedling can be considered an alternative to invasive surgical procedures in the reconstruction or regeneration of the missing interdental papilla [39].

The study by Gokhale et al. focuses on the relationship between plasma ascorbic acid levels and periodontal disease. The plaque index was used to evaluate the oral health, sulcus bleeding index, and probing pockets. According to the results, nonsurgical treatment combined with VitC intake significantly reduced the sulcus bleeding index of patients with gingivitis but had no further impact on the improvement of the clinical parameters of periodontitis [40]. Another study among adults showed that inadequate dietary VitC intake was associated with a 1.16 times higher likelihood of developing periodontitis than in those with adequate dietary VitC intake. According to Lee et al., the highest quartile of VitC intake (>132.2 mg/day) had a significantly lower community periodontal index score than those in the lowest quartile (47.34 mg/day) [41]. Recent studies revealed that the intake of VitC below a median dosage in women (OR 1.66; 95% CI: 1.04–2.64) and in non-smokers (OR 1.49; 95% CI: 1.04–2.14) was also linked to periodontitis [42,43]. Additionally, research showed that higher serum VitC concentrations were linked with decreased odds ratios for severe periodontitis (OR 0.53; 95% CI: 0.42–0.68) [44]. Moreover, a cross-sectional study found that patients with Stage IV periodontitis have significantly lower VitC levels than those in the early stages of periodontitis. Similar results were reported at Westmead Centre of Oral Health Periodontic Clinic, which showed that low VitC was associated with a higher periodontal disease stage (*p* = 0.03) [45]. As a result, low VitC serum levels may be regarded as a risk factor for periodontitis [46].

Furthermore, other studies investigated the effect of VitC on periodontal health and discovered an association between VitC supplementation and wound healing in patients with periodontitis after dental implant surgery. Li et al. showed that oral VitC supplementation improves postoperative healing after dental implant surgery in patients with chronic periodontitis and those treated with guided bone regeneration (GBR) or bio-oss collagen grafts [47].

**Figure 1:**
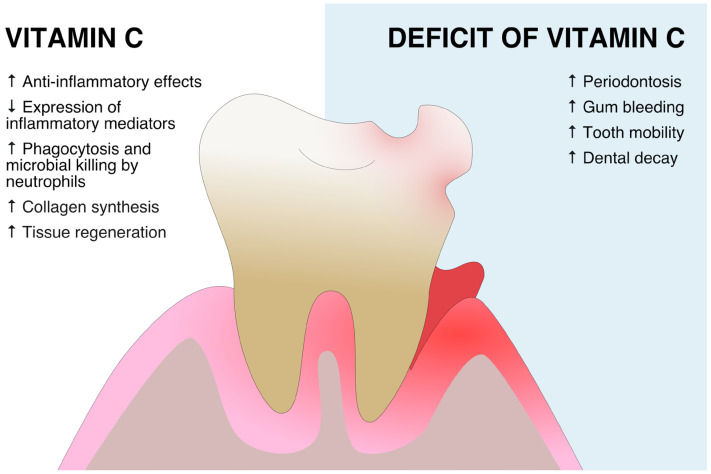
The role of vitamin C and possible effects of its deficiency on the periodontal tissue.

The plausible effects and most important studies regarding the role of VitC in periodontal disease are presented in Figure 1 and Table 1.

## 3. Vitamin D in Periodontitis

VitD is transported in the bloodstream as two main molecules: 25(OH)D3, created in the liver from VitD3 form, and a 1,25-(OH)2D3 form, created by 1α-hydroxylase, found mostly in kidneys, but also, for example, in the central nervous system (CNS) [48,49,50]. Its main function is calcium and phosphate homeostasis, although it also acts as an autocrine and paracrine agent. It binds to cells via a VitD receptor (VDR), which then modulates the expression of various genes and cell reactions. It is responsible for cartilage and bone matrix mineralization, and it helps to lock teeth in alveolar bone by strengthening the periodontal tissue. VitD deficiency in children is responsible for rickets, as low serum VitD levels accelerate bone turnover with subsequent bone demineralization causing skeletal deformities. As VDR receptors are also present in immune system cells, VitD has been shown to activate enhanced hydrogen peroxide secretion in monocytes and stimulate the synthesis of antimicrobial peptides such as β-defensin and cathelicidin. VitD also inhibits the proliferation of T lymphocytes and the transformation of B lymphocytes into plasma cells, leading to a decrease in the secretion of post-inflammatory cytokines and immunoglobulins. Studies suggest that through these mechanisms, VitD exhibits anti-inflammatory effects in periodontal tissue [51]. Additionally, VitD enhances macrophage phagocytic and chemotactic response and controls the 1-a-hydroxylase in monocytes, responsible for VitD creation as an autocrine agent, further accelerating immune response, being a critical part of periodontal tissue response to dysbiotic bacterial infection [51]. Regarding inflammation, VitD promotes the downregulation of proinflammatory cytokines. It has been shown that VitD can lower T-helper (Th) cells’ production of cytokines such as interleukin-17, which has been associated with an increased incidence of periodontitis [52]. All those factors make VitD a crucial agent in maintaining an organism’s homeostasis [17,53,54,55]. Nowadays, an increase in the need for VitD supplementation is seen, as more and more people tend to have less and less outdoor exercise and overall time spent exposed to sunrays [50]. This causes VitD deficiency, with the newest data showing that as much as 7% of the global population may be living with severe VitD deficiency, with a further 30% of the population having mild VitD deficiency [56]. Oral supplements mostly use the VitD3 form, which is absorbed by the intestinal wall and later transported in chylomicrons via the lymph system. However, to overcome malabsorption problems, a new form of calcifediol, a 25-hydroxyvitamin D (25(OH)D), has been created as an oral supplement. This form is transported via a portal vein with immediate access to the circulation [56]. The calcifediol form was shown to have an almost 100% absorption rate, and its serum levels increased linearly with the escalating administrated dose [56]. Sun exposure, if possible, is a great way to increase VitD levels. Nutritional diet is also essential because recent studies showed that highly processed foods with a low level of micronutrients stimulate gingival and periodontal inflammation. However, a diet rich in nutritional values (such as microelements, vitamins, complex carbohydrates, etc.) promotes healthy periodontium and overall health [6,57]. What is more, chronic inflammatory diseases (e.g., inflammatory bowel disease, cardiovascular diseases, and autoimmune disorders) may also promote the onset and continuation of periodontitis [58]. If ultraviolet radiation is scarce or the lifestyle does not promote VitD synthesis in vivo, supplementation comes as an almost natural choice.

VitD deficiency can lead to the periodontal inflammation process called periodontitis. VitD is present in saliva, and its levels are related to the VitD plasma concentration level. Constantini et al. showed that a low VitD saliva level was associated with an increase in biomarkers in patients with periodontitis [59]. A study by Taskan and Gevrek showed that periodontitis patients had lower levels of VDR with fewer fibroblast cells compared to a healthy group [60]. VitD’s anti-inflammatory and pro-mineralization effects on periodontal tissue have been shown in a recent in vitro study [61]. Periodontitis is also stimulated by oral microbiome alterations that worsen inflammation. VitD was shown to decrease the number of live *Porphyromonas gingivalis* sp. by stimulating their active autophagy [62]. Other studies show that high VitD levels decrease inflammatory levels of molecules (e.g., RANKL, interleukin-1, or interleukin-6) [63,64,65]. It was also proven that VitD is a vital molecule for maintaining tooth mineral density, as well as bone structure. Moreover, VitD concentration was reversely correlated with periodontitis severity [66,67,68].

In the case of periodontitis, the treatment consists of surgical and nonsurgical approaches. The nonsurgical way consists of anti-inflammatory drug administration and microinvasive treatment, such as probing the periodontium. Various compounds, such as ozonized hydrogel or chlorhexidine gel, can also be used for antibacterial effects [69,70]. Another viable option is antimicrobial photodynamic therapy, where light-sensitive materials are used to produce concentrated free radicals which are toxic for the bacteria. Recently, the use of probiotics has become the target of several studies, as researchers suggest that promoting the growth of non-pathogenic bacteria can prevent plaque formation. A study by Teughels et al. showed that using specific Streptococcus species, which are health-friendly bacteria, recolonization of periodontal pockets reduced inflammation of periodontal tissues [71]. The use of probiotics is also possible, as they stimulate the selective growth of host-beneficial bacteria in periodontal tissue [72,73]. Butera et al. evaluated nonsurgical methods of treating periodontitis. The results showed that the best result was observed with the use of a laser on periodontal tissues in peri-implantitis. The use of probiotics was beneficial in two of the six studies, while no statistical improvement was found for chlorhexidine and ozone [70].

The surgical approach uses surgical methods to reveal the periodontal space and manually remove the infected tissue [60,74,75]. The National Health and Nutrition Examination Survey III (NHANES III) study performed in the USA focused on the correlation between VitD levels and bleeding after probing. They showed that patients with the highest levels of VitD had experienced less bleeding when compared to those with the lowest levels of VitD, with a difference of up to 20% [76]. VitD influence on periodontium health was further investigated by other studies that found a correlation between VitD concentration and gingival inflammation [77]. It was also found that VitD and calcium supplementation showed moderate positive effects after nonsurgical treatment. Even though supplementation may help with the treatment of periodontitis, the study by Bashutski et al. proved that a low baseline level of VitD in patients had a negative impact on surgical treatment outcomes, despite later supplementation [78]. When surgical treatment is not sufficient, tooth extraction is needed as the periodontium and gingival tissue is weakened by the inflammation. In several studies, it was shown that low VitD levels were correlated with a higher risk of tooth loss [79,80,81]. VitD was also proven as a statistically significant molecule for maternal periodontal health. The severity of periodontitis among pregnant women was linked to lower serum levels of VitD [82,83,84]. However, some of the studies showed inconsistent results with VitD levels, which need further evaluation [85,86,87].

**Figure 2:**
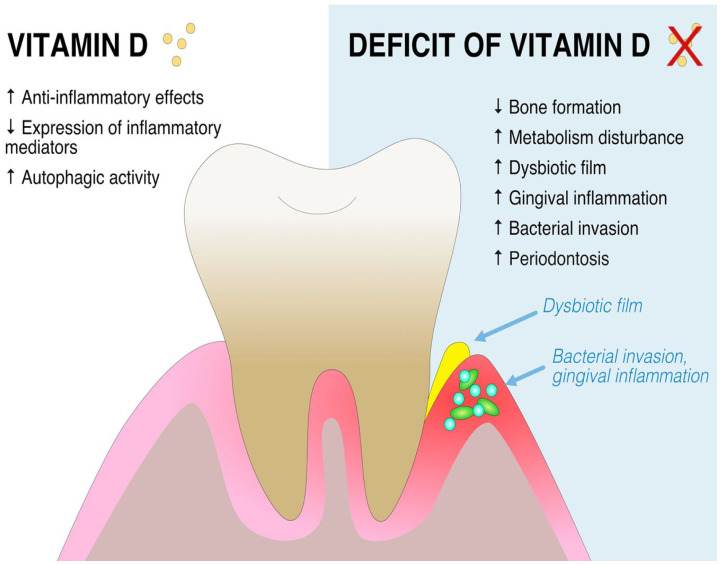
The role of vitamin D and possible effects of its deficiency on the periodontal tissue.

Naturally, VDRs come in polymorphism variants, making VitD supplementation quite a challenge. VDR variants come with different VitD bonding capacities, as shown in different studies [88,89,90]. VitD has an essential role in periodontitis, as a sufficient VitD level has beneficial effects on periodontitis prevalence and severity. The meta-analysis by Wan et al. showed that some VDR variants have a high correlation with periodontitis [89]. They focused on the four most common VDR polymorphisms, FokI (rs2228570), ApaI (rs7975232), TaqI (rs731236), and BsmI (rs1544410). The BsmI polymorphism is suggested to have a high correlation with periodontitis under the recessive model (OR = 0.722, 95% CI: 0.532–0.980, *p* = 0.037). The FokI polymorphism has a high correlation in a dominant and allelic model (OR = 1.459, 95% CI: 1.050–2.028, *p* = 0.025; OR = 1.386, 95% CI: 1.026–1.874, *p* = 0.034, respectively). ApaI and TaqI polymorphisms showed no statistical correlation for chosen populations. Another meta-analysis by Yu et al. agrees that the FokI polymorphism has a high correlation with periodontitis prevalence [88]. As novel VDR polymorphisms are being found, more data are becoming available to researchers, resulting in promising studies being carried out [51]. A summary of VitD effects on periodontal tissues is presented in Figure 2. A summary of the most important studies on the effects of VitD on periodontal health is presented in Table 2.

## 4. Discussion and Conclusions

Periodontitis is a widespread disease caused by numerous factors, such as dysbiotic microbiome, lack of oral hygiene, stress, smoking, malocclusions, and improper diet. Genetically predisposed individuals could develop exacerbated symptoms [91]. Underlying the development of periodontal disease is primarily an immune response triggered by infectious microorganisms located within the oral microflora, particularly the subgingival plaque. Research indicates that both bacterial biofilm and periodontal inflammation play an essential role in the onset and development of periodontal disease and reinforce each other [10,12,55,61]. Pathogenic microorganisms localized in the periodontal plaque produce enzymes and other substances that cause the development of chronic inflammation in the periphery of the periodontal tissues, leading to their damage. Within the periodontal tissues, several processes are activated, in the development of which numerous mediators are involved, such as proinflammatory cytokines and chemokines and free radicals [4,5,6]. Research in recent years indicates that vitamins such as VitC and VitD are also involved in these processes. The newest research suggests that promising treatment strategies for periodontal disease should be based on therapies that target all molecular and cellular causes of disease development [69]. Despite the progress that has been made in the knowledge of the causes of periodontal disease, there is still a need to deepen our understanding of its pathogenesis. It is particularly important to know the mechanisms that regulate homeostasis in periodontal tissues and the factors that disrupt it. It is important to know the contribution of the balance between oxidants and antioxidants to the development of the inflammatory process. Only by addressing all factors contributing to the development of periodontitis can periodontitis be successfully treated and long-term remission be achieved.

Periodontitis treatment is a complex matter that requires a broad range of interventions to control and decrease inflammation. The most crucial step of the therapy is the regulation of the oral biofilm, both by periodontist intervention and patient healthy habits. Its exacerbation is associated with dental plaque, overgrowth of the subgingival biofilm, and calculus build-up. Other proven risk factors must also be supervised and controlled. As such, a healthy and nutritionally rich diet is also essential. Periodontal inflammation can be further reduced by nonsurgical therapy, vitamins, prebiotics, probiotics, and symbiotic agents [70,92].

In this article, we focused on several studies evaluating the association of vitamins C and D with their effect on periodontitis. VitC has an inhibitory effect on oxidative stress and the synthesis of proinflammatory mediators, while VitD has an inhibitory effect on the secretion of proinflammatory cytokines [33,63]. This influence on the inflammatory process could have a beneficial effect on periodontitis.

Moreover, VitC and VitD reduce bleeding within the gums, which can be used in additional therapies for periodontal disease. This may increase patient quality of life, alleviate symptoms, and enable proper oral hygiene in people affected by the disease. Low concentrations of both vitamins in the serum correlate with the progression of the disease and increase the risk of tooth loss [81]. VitC and VitD seem to be essential elements in preventing periodontitis [60]. Fibroblasts are found in both the skin and periodontal tissues. Their reduced number within the periodontal fibers is associated with reduced stabilization of the tooth in the alveolus and impaired wound healing. L-ascorbic acid is widely used in dermatology as a stimulator of fibroblast proliferation [93]. The antioxidant and protective effect of VitC on periodontal cells is also noteworthy [38], meaning that VitC can be used as a potential factor in protecting periodontal ligament cells.

The importance of supplementation and a healthy diet is emphasized in many articles related to dental diseases and may benefit the whole body. VitC and VitD were reported to impact the pathogenesis of periodontitis, leading to alterations in the structure of periodontal ligaments. VitC is a significant nutrient involved in numerous physiological processes. It has a crucial role in the collagen stabilization process, resulting in participation in collagen enzymatic transformation. It was reported that VitC could have a protective effect on periodontal tissues and could decrease levels of proinflammatory cytokines. An inadequate intake was also associated with a higher risk of periodontal disease. Studies showed that patients with periodontitis have a lower level of VitD receptors and a lower number of fibroblast cells compared to healthy patients. Furthermore, studies showed the anti-inflammatory and pro-mineralization effects of VitD. Moreover, a high VitD level decreases levels of inflammatory molecules, and VitD is an important molecule for maintaining tooth mineral density and bone structure. VitD concentration was found to be inversely related to periodontitis severity. It was also found that VitD and calcium supplementation showed moderate positive effects after nonsurgical treatment. Both vitamins may decrease gum bleeding, which can be used to improve patient quality of life. A growing number of studies suggest that adequate VitC and VitD intake could have a beneficial effect on both the prevention and treatment of periodontal disease. However, further research regarding the role of vitamins is crucial to understanding their effect on periodontal tissues. This could lead to the development of novel, promising therapeutical options for periodontitis.

The purpose of this review was to summarize studies evaluating the potential role of vitamins C and D in the pathogenesis and treatment of periodontitis. However, our review has some limitations. The research design was retrospective, based on many publications from PubMed, Google Scholar, and Web of Science. Our goal was to find the most valuable and widely studied papers. They were selected taking into account a number of criteria, such as the authors’ experience in the field, scientific achievements, and innovations. Therefore, additional relevant studies might have been missed. Secondly, the number of articles for the review is limited. Nevertheless, the articles reviewed in this study showed an association between vitamin C and D and periodontitis. The results of previous studies indicate that vitamins C and D have a substantial impact on the proper functioning of periodontal tissues and the development of periodontitis when they are deficient. A thorough understanding of the effects of vitamins C and D on the development of periodontitis allows their use in the pharmacological treatment of this disease, while there is a need for further research in this area.

## Figures and Tables

**Table 1:** A summary of the most important studies on the effects of VitC on periodontal health.

Study	Sample	Control	Measured Variable	Assessment Method	Results
N.H. Gokhale, A.B. Acharya, V.S. Patil, D.J. Trivedi, S.L. Thakur (2013) [40]	90 cases with different stages of periodontitis	30 healthy individuals	Serum VitC levels with sample groups receiving VitC supplementation after first clinical examination	Plaque index (PlI), sulcus bleeding index (SBI), and probing pocket depths	Serum VitC levels were lower in sample groups compared to the control group. Subsequent supplementation of VitC reduces the SBI score.
J.A. Park, J.H. Lee, H.J. Lee, B.H. Jin, K.H. Bae (2017) [42]	2049 young adults aged 19–39 years divided into groups based on clinical examination and nutrition survey		Vitamin and mineral intakes	Community Periodontal Index (CPI)	There were significant associations of periodontitis with lower intake of vitamin C in women (OR 1.66; 95% CI 1.04–2.64) and in current non-smokers (OR 1.49; 95% CI 1.04–2.14).
I.L. Chapple, M.R. Milward, T. Dietrich (2007) [44]	Analysis of 11,480 NHANES III adult participants (>20 y of age).		Serum VitC level	Severity of periodontal disease	Increased serum antioxidant concentrations are associated with a reduced relative risk of periodontitis of 0.53 (95% CI, 0.42, 0.68) for vitamin C.
M. Assaf, H. Rabi (2022) [46]	25 patients with different stages of periodontitis		Serum VitC level	Clinical examination	Severity of periodontitis was inversely proportional to VitC serum levels (*p* < 0.05).
X. Li, L. Tang, Y.F. Lin, G.F. Xie (2018) [47]	128 patients requiring dental implants divided into an experimental subgroup, who received vitamin C, and a control subgroup		Soft tissue healing and pain response scores	Clinical examination	The experimental subgroups had significantly higher healing indices than the controls (*p* < 0.05).
M.R. Munday, R. Rodricks, M. Fitzpatrick, V.M. Flood, J.E. Gunton (2020) [45]	20 patients with periodontitis		Serum VitC and C-reactive protein (CRP) levels	Clinical examination	Low VitC was associated with higher periodontal disease stage (*p* = 0.03). Elevated CRP was found in 2/3 of people with low VitC, and CRP was negatively correlated with VitC (*p* < 0.01).

**Table 2:** A summary of the most important studies on the effects of VitD on periodontal health.

Study	Sample	Control	Measured Variable	Assessment Method	Results
G.N. Antonoglou et al. (2015) [80]	55 cases of chronic periodontitis	30 healthy individuals	Serum VitD level	Plaque index	Low 1,25(OH)2D was correlated with a risk
K.A. Boggess et al. (2011) [82]	117 cases of maternal periodontitis	118 healthy pregnant women	Serum VitD level	Clinical symptoms of moderate to severe periodontitis	Pregnant women with periodontitis had a median serum VitD concentration of 59 vs. 100 nmol/L in healthy group (*p* < 0.001)
W. Gao et al. (2020) [75]	120 cases of moderate to severe periodontitis after surgical treatment with 1000 IU/d VitD supplementation and 120 cases of 2000 IU/d VitD supplementation	120 cases of periodontitis after surgical treatment without VitD supplementation	VitD supplementation of none, 1000 IU/d, and 2000 IU/d	Clinical examination	Groups with VitD supplementation had a significant decrease in attachment loss and probing depth compared to the control group
J. Han et al. (2019) [63]	Rat model for periodontitis with experimental group receiving VitD intraperitoneally vs. control group being treated with peanut oil		RANKL, TNF-α and interleukin serum levels	Examination of inflammatory status	VitD treatment alleviates inflammation by decreasing serum levels of RANKL, TNF-α, and interleukin-1 and increasing interleukin-10 levels
J.L. Ebersole, J. Lambert, H. Bush, P. E. Huja, A. Basu (2018) [81]	Three NHANES cohort studies with 15,844 adults with periodontal examination, divided into healthy population and periodontitis population		Serum VitD level	Periodontal examination	Lower levels of VitD were seen in periodontitis population vs. healthy population
G. Isola et al. (2020) [77]	46 periodontitis cases	43 healthy patients	Serum VitD level	Periodontal examination	Periodontitis group had a significantly lower mean concentration VitD levels (17.4 ± 5.2 ng/mL) vs. healthy group (29.9 ± 5.4 ng/mL) (*p* < 0.001)

## Data Availability

Not applicable.
